# Clinicians’ perceptions of family involvement in the treatment of persons with psychotic disorders: a nested qualitative study

**DOI:** 10.3389/fpsyt.2023.1175557

**Published:** 2023-05-24

**Authors:** Lars Hestmark, Maria Romøren, Kristiane Myckland Hansson, Kristin Sverdvik Heiervang, Reidar Pedersen

**Affiliations:** ^1^Centre for Medical Ethics, Institute of Health and Society, University of Oslo, Oslo, Norway; ^2^Department of General Practice, Institute of Health and Society, University of Oslo, Oslo, Norway; ^3^Division of Mental Health Services, Akershus University Hospital, Lørenskog, Norway; ^4^Faculty of Health and Social Sciences, Center for Mental Health and Substance Abuse, University of South-Eastern Norway, Drammen, Norway

**Keywords:** family involvement, psychotic disorders, family psychoeducation, qualitative methods, mental health services research

## Abstract

**Background:**

Family involvement in mental health care ranges from basic practices to complex interventions such as Family psychoeducation, the latter being a well-documented treatment for psychotic disorders. The aim of this study was to explore clinicians’ perceptions of the benefits and disadvantages of family involvement, including possible mediating factors and processes.

**Methods:**

Nested in a randomised trial, which purpose was to implement Basic family involvement and support and Family psychoeducation in Norwegian community mental health centres during 2019–2020, this qualitative study is based on eight focus groups with implementation teams and five focus groups with ordinary clinicians. Using a purposive sampling strategy and semi-structured interview guides, focus groups were audio-recorded, transcribed verbatim, and analysed with reflexive thematic analysis.

**Results:**

Four main themes were identified as perceived benefits: (1) Family psychoeducation—a concrete framework, (2) Reducing conflict and stress, (3) A triadic understanding, and (4) Being on the same team. Themes 2–4 formed an interconnected triad of mutually reinforcing elements and were further linked to three important clinician-facilitated sub-themes: a space for relatives’ experiences, emotions and needs; a space for patients and relatives to discuss sensitive topics and an open line of communication between clinician and relative. Although far less frequent, three main themes were identified as perceived disadvantages or challenges: (1) Family psychoeducation—occasional poor model fit or difficulties following the framework, (2) Getting more involved than usual, and (3) Relatives as a potentially negative influence—important nonetheless

**Conclusions:**

The findings contribute to the understanding of the beneficial processes and outcomes of family involvement, as well as the critical role of the clinician in achieving these and possible challenges. They could also be used to inform future quantitative research on mediating factors and implementation efforts.

## 1. Introduction

Persons with psychotic disorders may experience positive symptoms, such as hallucinations and delusions, and negative symptoms, such as social withdrawal, emotional apathy, and lack of drive. These symptoms may be accompanied by reduced functioning, cognitive impairment, and altered behaviour (1), affecting the life and well-being of both patients and their relatives (2). In this study, we use the terms ‘family’ and ‘relatives’ to describe anyone who provides considerable and unpaid support to a person with a psychotic disorder. ‘Family involvement’ is an umbrella term that covers any systematic practice to include relatives in the assessment, treatment, and follow-up of the patient, but also efforts to address the needs of relatives themselves.

There is a continuum between basic family involvement practices and the more complex models that are referred to as family interventions (3). It is vital to establish contact and alliance with relatives, listen to their experiences and concerns, assess their strengths, limitations, burdens, and needs and provide them with general information about the illness, treatment, health services, and available support measures. Relatives may also provide clinicians with important collateral information, contribute to the development of a crisis/coping plan, and alert the health services when the patient’s symptoms worsen. This basic level of family involvement and support is a necessary foundation for family interventions, which have become a pillar of the evidence-based treatment for psychotic disorders.

The various family interventions used in mental health care have much in common, even if based on different theoretical assumptions (4). The label ‘Family psychoeducation’ (FPE) is applied to a group of widely used and well-documented models that can be offered in a single- or multi-family format. These grew out of the realisation that schizophrenia is not caused by ‘pathological’ families, as was previously assumed. Rather, the high levels of ‘expressed emotion’ (EE) in some families, consisting of hostility, criticism, and emotional over-involvement, may reflect their attempt to deal with the patient’s illness, often without sufficient knowledge, understanding, and coping skills (5). Evidence suggest that a high level of EE may further increase the risk of relapse, in accordance with the stress-diathesis model (6, 7). The FPE models target this vicious circle, by having clinicians provide both patient and relatives with emotional support, information concerning the illness and treatment, coping skills, recognition of warning signals, communication skills and structured problem-solving (5, 8).

Research shows that family interventions in general may improve the function, quality of life and adherence with treatment for persons with psychotic disorders, while also reducing the number of relapses and the number and length of hospital admissions (9–12). For relatives, these interventions may improve their experience of caregiving, their quality of life and the family function, as well as reduce their carer burden, distress and the level of EE (13–16).

The mediating factors and processes that generate these beneficial effects are of major interest, to identify core elements and improve the existing models (17). In this context, qualitative methods can be used to investigate the dynamics of family involvement to generate hypotheses for quantitative research. Some qualitative studies have explored the benefits and dynamics of FPE and similar models from patients and relatives’ viewpoint (18–23), whereas studies on clinicians’ experiences have largely focused on barriers and challenges (24, 25). In addition, qualitative studies have investigated basic family involvement practices as an integrated part of inpatient wards (26, 27), early intervention services (28, 29) and assertive outreach teams (30). However, there is a need for qualitative studies exploring how clinicians’ perceive the utility and processes of FPE, as well as studies investigating combinations of basic family involvement practices and family interventions.

This qualitative study was nested in a cluster randomised trial, which purpose was to implement guidelines on family involvement for persons with psychotic disorders in Norwegian community mental health centres (CMHCs) (31). These national guidelines recommend both basic family involvement practices and family interventions (32, 33). A qualitative evaluation of the implementation process found that practicing family involvement was a major facilitator for implementation, since witnessing its benefits first-hand inspired the clinicians to continue (34). The present article follows up on this topic and aims to explore clinicians’ perceptions of the utility of family involvement, including possible mediating factors and processes, by answering the following research question: how did mental health professionals experience using family involvement in the treatment of persons with psychotic disorders, in terms of perceived benefits and disadvantages for patients, relatives and clinicians?

## 2. Methods

This article is written in accordance with the ‘Standards for Reporting Qualitative Research (SRQR)’ (35) (Supplementary material 1).

### 2.1. Study design, context, and interventions

The cluster randomised ‘Implementation of Family Involvement for persons with Psychotic disorders’—(IFIP) trial (31) took place in South-East Norway. Fourteen CMHC clusters were allocated to the experimental or control arm, whereupon the seven experimental clusters received an implementation support programme to implement national guidelines on family involvement from July 2019 to the end of 2020. The clinical units in both arms varied significantly in terms of size, geographical location, service type, and patient population (36). The study has been approved by the Norwegian regional committee for medical and health research ethics (REC) South-East with registration number 2018/128.

The Norwegian Directorate of Health has published national recommendations on family involvement and support in the health and care services, based on legal regulations, research evidence, ethical considerations, and discussions between key stakeholders and experts (33). These include general recommendations on identifying relatives, clarifying their role, and documenting the relevant information in the medical record, and further on how to involve relatives in the assessment, treatment, and follow-up of the patient, while supporting them during various phases of the patient’s illness. The recommendations were condensed and operationalised as part of the IFIP project to produce a clinical intervention called ‘Basic family involvement and support’ (BFIS) (31). The Directorate of Health has also issued clinical practice guidelines that recommend FPE specifically in the treatment of psychotic disorders during all phases of the illness (32). Consequently, the clinical interventions of the IFIP trial included both BFIS and FPE, which overlap to some extent (Table 1).

**Table 1:** Clinical interventions of the IFIP trial.

1. Basic family involvement and support	At least three conversations about family involvement: one conversation with the patient alone, one with the relative(s) alone and one joint conversation
	Written information about the family involvement at the unit, web resources and available support measures
	Psychoeducative seminars for relatives
	Developing a crisis/coping plan
2. Family psychoeducation (FPE) in single-family groups	Engagement and alliance sessions
	Warning signals, crisis/coping plan, genogram, goals of treatment
	Psychoeducation
	Communication skills exercises
	Problem-solving sessions

The IFIP trial employed multiple implementation strategies and interventions on both organisational and clinical levels. An important measure was the establishment of local implementation teams to plan and oversee the implementation effort. The teams usually included the local leader(s), an appointed family coordinator, one or more clinicians, and preferably also a user representative. One of the clusters in the experimental arm consisted of two clinical sites, each of which had its own implementation team. All clinicians in the experimental clusters were offered training and supervision in FPE and BFIS. They were encouraged to offer BFIS to all patients and their relatives, and FPE to as many of them as possible (37).

### 2.2. Sampling, participants and data collection

During the IFIP trial, implementation teams (*n* = 8) were interviewed two times, in the start and middle phases of the implementation period, whereas groups (*n* = 5) of ordinary clinicians were interviewed in the late phase. At three of the units, we chose to conduct focus groups with the implementation teams only, because these included a majority of the units’ clinicians. For this particular study, the data material only included the second round of focus groups with the implementation teams, as well as the focus groups with ordinary clinicians, since implementation team members had not gained sufficient experience with the intervention in the early phase of the trial. Table 2 provides an overview of the participants.

**Table 2:** Overview of participants in focus groups with implementation teams and ordinary clinicians during the middle and late phases of the IFIP trial.

	Implementation team members		Ordinary clinicians	
	Middle phase of the trial		Late phase of the trial	
	January/February 2020 (*N* = 39; 8 focus groups)		September/October 2020 (*N* = 25; 5 focus groups)	
	*N*	%	*N*	%
**Sex**				
Male	5	13	5	20
Female	34	87	20	80
**Age (years)**				
20–35	5	13	7	28
36–50	16	41	11	44
51–70	18	46	7	28
**Prof. background/role**				
Section/unit manager	5	13		
Physician	3	8	4	16
Psychologist	5	13	16	64
Psychiatric nurse	15	38	1	4
Other	11	28	4	16

The sampling strategy was purposive, aiming to interview clinicians who had practised systematic family involvement in the treatment of patients with psychotic disorders. We expected the implementation team members to be particularly dedicated and positive, whereas focus groups with ordinary clinicians could provide us with complementary and perhaps even critical perspectives. The latter were recruited through the local leaders according to our specific instructions: groups had to consist of 3–6 participants with various professional backgrounds, who could not be leader(s) or members of the implementation team. They must have practised family involvement for this particular patient group and at least one of them must have provided an entire course of FPE. We also encouraged the local leaders to include participants who were sceptical of, or less committed to, FPE or family involvement in general.

We obtained written informed consent from all participants before the start of each focus group. Using semi-structured interview guides (Supplementary material 2), most focus groups were carried out by two researchers visiting the site in question. Because of restrictions during the coronavirus pandemic, three of the focus groups were conducted with only one researcher being present. Participants were asked about the significance and utility of family involvement for the various stakeholders, including positive and negative experiences. They were also asked about ethical dilemmas and conflicts of interest, specifically concerning information sharing and confidentiality. Focus groups lasted for 60–90 min, were audio-recorded and transcribed verbatim. Recordings, transcriptions and field notes were stored in the University of Oslo’s secure database (TSD). The resulting data material has previously been analysed to explore barriers and facilitators when implementing family involvement (34), as well as challenges related to confidentiality and information sharing (38).

### 2.3. Data analysis

Using a realist inductive approach to identify themes mainly at a semantic level, the first author employed Braun and Clarke’s method for reflexive thematic analysis (39, 40). There were no strict criteria for how frequent a pattern must be identified to constitute a theme. However, themes must be identified in focus groups with both implementation teams and ordinary clinicians, and must not be based solely on two focus groups from the same unit. All the data material was given equal attention in the coding process, but the analysis was focused and guided by the research question. The NVivo 12 software was used to store, organise, and code the data.

In addition to following the six phases described by Braun and Clarke (39), we added the following steps: the initial coding and thematic map was discussed with the co-authors to see if there were other ways of reading and interpreting the data. Preliminary themes and thematic maps were also discussed with the project’s stakeholder committee, with valuable input both on themes that were already identified and on other possible themes. One of the co-authors simultaneously analysed interviews with patients, guided by a similar research question, and the results from both analyses were compared to look for similarities between clinicians and patients’ perspectives. Thus, trustworthiness was enhanced by investigator triangulation (including both researchers and stakeholder representatives), and by data triangulation (including two kinds of focus groups with different participants in the analysis, as well as comparing the findings with those from interviews with patients). The results are presented below as a combination of condensed text and illustrative quotes.

### 2.4. Reflexivity

We are aware of the embedded and non-neutral position of all the authors of this article, as researchers who assisted and promoted the implementation of family involvement at the clinical sites where the participants worked. Consequently, we have strived to elicit critical perspectives in the focus groups and to provide a comprehensive account of clinicians’ experiences.

## 3. Results

**Figure 1:**
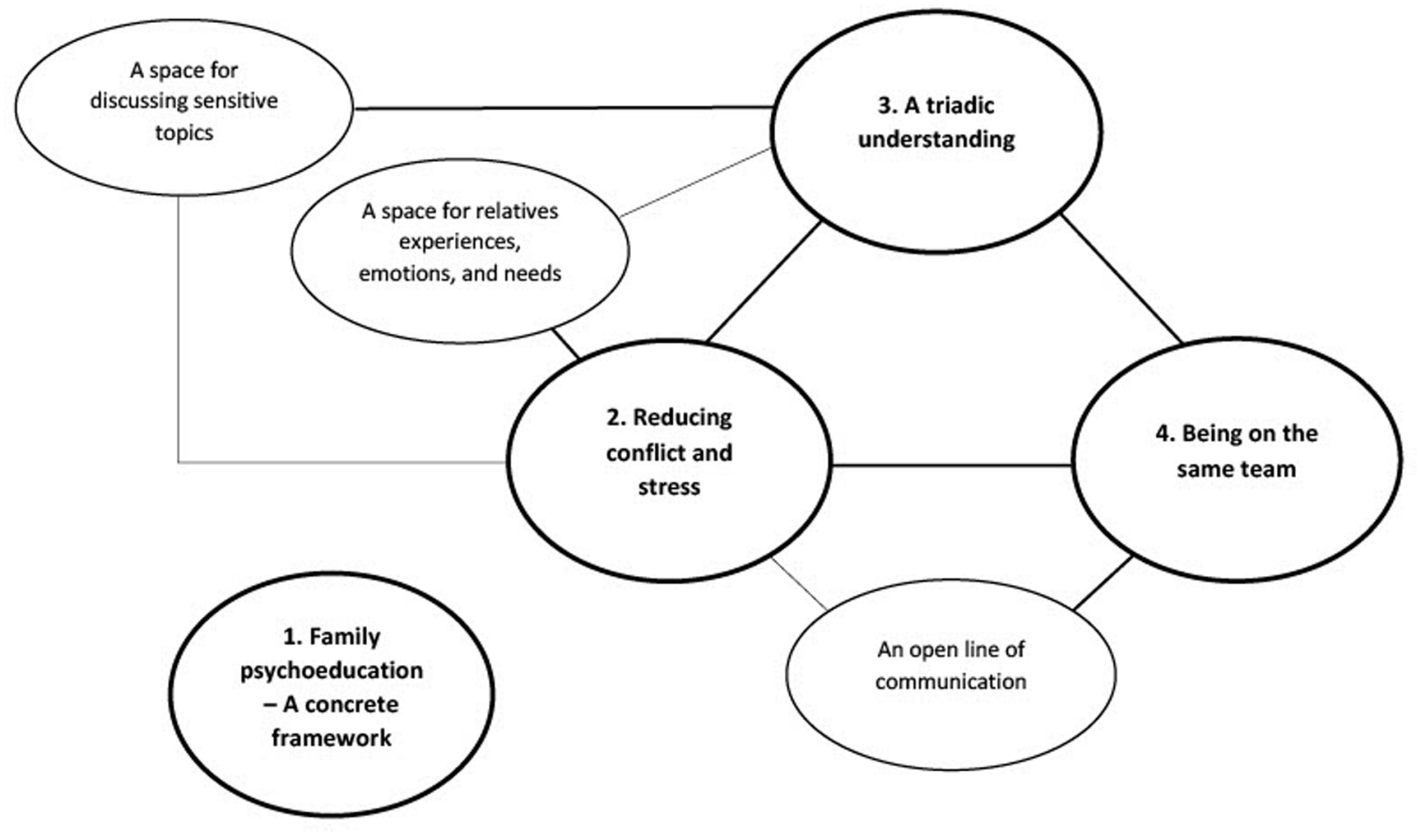
Thematic cluster 1: perceived benefits of family involvement, based on thematic analysis of focus groups with implementation team members and ordinary clinicians during the IFIP trial.

We identified four main themes that were categorised as perceived benefits (Figure 1): (1) Family psychoeducation—a concrete framework, (2) Reducing conflict and stress, (3) A triadic understanding, and (4) Being on the same team. Theme 1 described clinicians’ overall perceptions of the FPE model and its structure, whereas themes 2–4 concerned the content, processes and utility of both BFIS and FPE, forming an interconnected triad of mutually reinforcing elements. Themes 2–4 were further linked to three important clinician-facilitated sub-themes: a space for relatives’ experiences, emotions and needs; a space for patients and relatives to discuss sensitive topics and; an open line of communication between clinician and relative.

**Figure 2:**
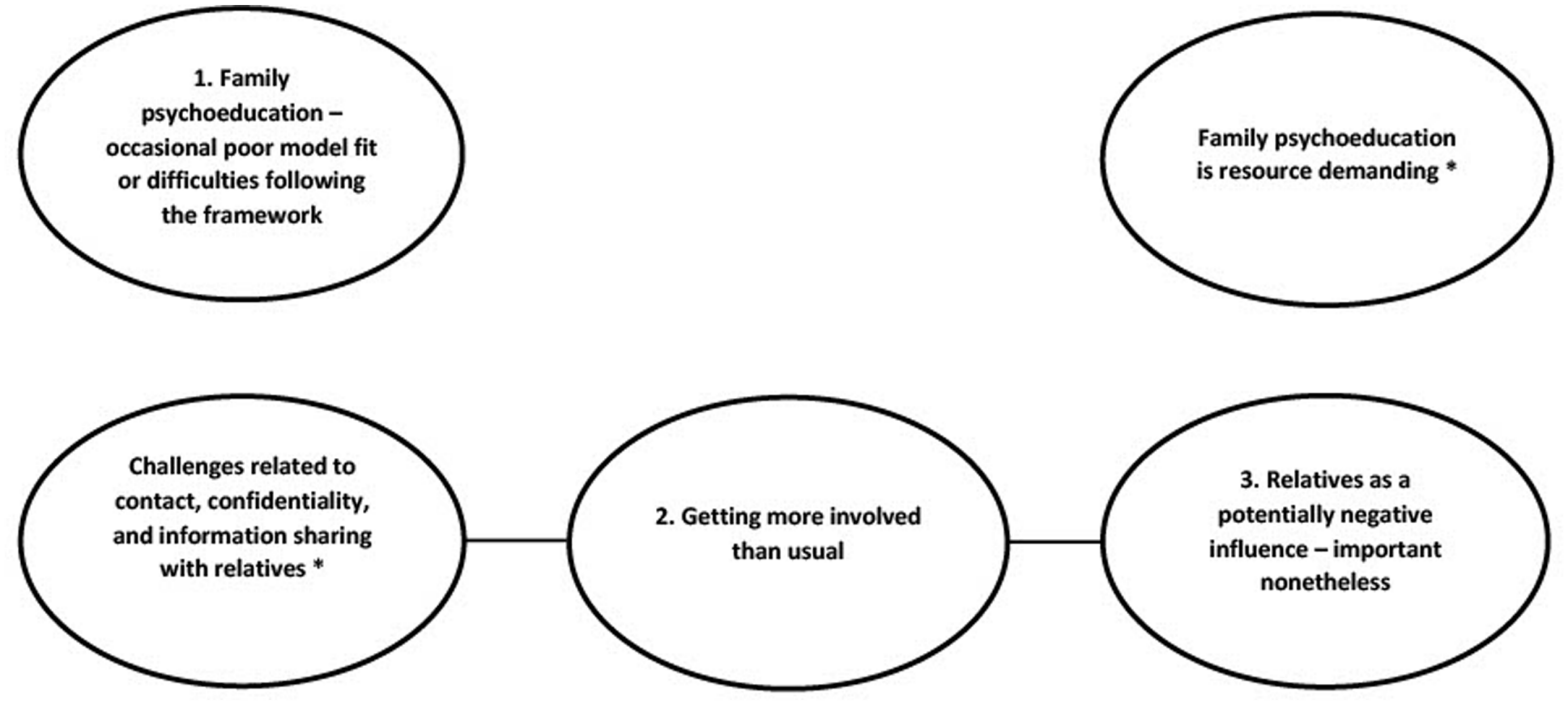
Thematic cluster 2: perceived disadvantages or challenges of family involvement, based on thematic analysis of focus groups with implementation team members and ordinary clinicians during the IFIP trial. *Main themes that have been analysed in previous articles.

Concerning perceived disadvantages or challenges, we identified three main themes (Figure 2): (1) Family Psychoeducation—occasional poor model fit or difficulties following the framework, (2) Getting more involved than usual, and (3) Relatives as a potentially negative influence—important nonetheless. These themes were reported much less frequently than the perceived benefits. However, to provide a comprehensive account of clinicians’ experiences, we have allowed their perceptions of disadvantages or challenges more space than their frequency would normally suggest.

Clinicians sometimes distinguished between BFIS and FPE, but usually shared their experiences of family involvement in general. The distinction is also blurred by the fact that they frequently used elements of FPE without offering the entire model, and that the initial phases of FPE are nearly identical to BFIS. When they attributed some benefit or disadvantage directly to either FPE or BFIS, we have emphasised this in our account. During the focus groups, clinicians used the terms ‘family’ and ‘relative’ broadly to refer to any significant person that had been involved in the assessment, treatment, and follow-up of the patient. There were no consistent thematic differences between the focus groups with implementation teams and those with ordinary clinicians. Illustrative quotes are labelled with ‘FG’ (Focus Group) followed by a number corresponding to a specific focus group.

### 3.1. Perceived benefits of family involvement

#### 3.1.1. Family psychoeducation—a concrete framework

Clinicians were enthusiastic about offering a concrete and evidence-based intervention that is recommended in the clinical practice guidelines. Some reported an increased satisfaction with their clinical work, describing family involvement as both developing and meaningful. They further observed that relatives and patients appreciated being offered something concrete, structured and useful, which involved long-term cooperation. Clinicians frequently referred to the FPE model as a tool, or set of tools, where briefer versions or single elements could be employed in various therapeutical contexts. The elements, such as the problem-solving structure, could also be used by patients and relatives at home.

**FG5:** ‘I am in the middle of one (FPE) course, and then I have started one such «light version». And so, conversations with relatives is something I have always had, but now it is more systematised and I do feel that it is very nice to have something concrete, a tool. And then be able to refer to it, it is slightly easier then to sell it to both patient and relatives.’

The standardised length, content and sequence of elements was experienced as a useful aid by many clinicians, helping the groups return to a constructive process when sidetracked. They also saw that structure ensured predictability for patients, who may suffer from cognitive impairment. At the same time, clinicians considered the model flexible enough to accommodate different types of families and family dynamics.

**FG6:** ‘(…) I experience that the tight structure, because there is room within the structure and… Right, to facilitate and also manage to deviate if there should be a reason for it, (…). And I am not afraid to do that, so I (…) also find that structure to be good. I see that for the patient it is important to be able to cope with being there.’

#### 3.1.2. Reducing conflict and stress

An overarching theme was that family involvement seemed to reduce conflict and stress. The conflicts described were usually between health personnel and relative(s) or between relative(s) and patient, while all stakeholders could experience stress. Conflict and/or stress often resulted from a lack of contact, cooperation and information exchange between relatives and health personnel, as well as a lack of openness and understanding between patient and relative. Family involvement, with the FPE model in particular, addressed these issues systematically and the results were described as ‘lowered shoulders,’ ‘calmer relatives and home environment,’ and ‘reduced nagging and critical comments.’ Clinicians further emphasised the utility of the ‘communication rules’ in FPE, and that family involvement could improve the communication between patient and relative(s).

An important sub-theme, linked to reducing conflict and stress, was to create a space for relatives’ experiences, emotions and needs. Earlier when talking with relatives the focus was usually on obtaining collateral information, but now clinicians also asked them how the patient’s illness affected their life and well-being. Relatives could ‘ventilate’ and articulate their frustration, without the patient being present and without clinicians judging them or defending the health services, but rather ‘containing’ their emotions by acknowledging and normalising them. This cathartic process appeared to greatly relieve their stress and reduce any resentment towards the health services, making it possible to start over and establish an alliance between clinicians and relatives. The alliance sessions in FPE emphasised this process specifically, but clinicians also reported using this competence outside the model. By focusing on relatives’ experience, situation and needs it was easier to offer them adequate information, guidance and support.

**FG7:** ‘(…) so a part of what I too experience that they (the relatives) appreciate is the validation of their own, what should I say, vulnerable topics. Things like one having done something wrong or that one is to blame for the patient becoming ill, and that they also get to hear that it is normal to have those thoughts, and receive psychoeducation about the illness (…) makes it easier for them to be relatives.’

**FG11:** ‘(…) they (the relatives) seem more secure, that is (I) notice that, that relatives may not be as eager to make demands or require information, but get a sense of security in that, «yes we have a space where we get what we need». And that it also results in lowered stress for the patient.’

#### 3.1.3. A triadic understanding

Clinicians observed that establishing contact and building an alliance with relatives, in addition to the patient-therapist alliance, opened up the possibility for a triadic understanding between clinician, patient and relative(s). The theme ‘triadic understanding’ includes an increased mutual understanding and acknowledgement, as well as a shared understanding. The latter term means sharing a platform of knowledge and concepts without necessarily agreeing on everything.

To create a space for discussing sensitive topics, characterised by trust, openness and a sense of equality between participants, was described as a critical foundation for a triadic understanding. It presupposed trust and alliance between all stakeholders, particularly between patient and clinician. Clinicians experienced that patient and relative(s) could discuss matters that were difficult to bring up in everyday conversation, perhaps because they were hard to articulate for the patient and/or were sources of conflict at home (such as substance abuse or negative symptoms). An important function of health personnel, referring to themselves in this context as ‘diplomats,’ was to put into words and explain to the relatives how the patient was feeling or experiencing the illness, on the patient’s request.

**FG1:** ‘If for instance a boy/girlfriend comes along then, so even if they talk together a lot, they tend not to talk about the things that are important, and that it is good to just have that space. To talk, talk together and that the next of kin get to know a bit more.’

Clinicians emphasised that psychoeducation was a joint effort to establish a shared understanding, where they employed concepts, descriptions and illustrations from the FPE manual that patients and relatives could recognise as relevant to their experience.

**FG10:** ‘(…) sometimes we asked (the patient), «Yes can you show us where you are on the didactic illustration? » And that is very good because then you speak the same language.’

A shared platform of knowledge and concepts, together with increased openness and a space to discuss sensitive topics, facilitated mutual understanding and acknowledgement. Clinicians observed that relatives gained an understanding of diagnosis and symptoms, particularly of negative symptoms, which further enabled them to understand and acknowledge the patient’s situation better, adjust their expectations and reduce critical comments. In addition, clinicians provided relatives with guidance and concrete measures to handle challenging situations in a supportive way. Thus, anxiety, stress and conflict at home was reduced and relatives appeared more competent and secure to deal with illness-related issues.

**FG7:** ‘(…) that the level of conflict within the family decreases. That it is both a question of solving various problems that often result in conflict, but perhaps in particular a different understanding of what is going on. That it is not a matter of laziness and things like that.’

**FG3:** ‘And understand (…) what they (the relatives)… What is sort of… Good things they can do themselves, when she is ill.’

Clinicians reported that relatives also gained an understanding of treatment, follow-up and prognosis, as well as the role of clinicians and the health services, which helped avert misunderstandings. Patients also seemed to appreciate relatives and clinicians’ perspectives to a larger degree, although clinicians brought this up less frequently.

One of the most profound changes among clinicians was how they came to acknowledge relatives’ situation and perspective through family involvement. This emerged as general reflections on relatives’ burdens, needs and motivations, as well as accounts of specific experiences where family involvement provided such insights. In several instances, they related this phenomenon directly to the alliance sessions of FPE.

**FG12:** ‘I do think that the alliance sessions are gold in relation to us really wishing them (the relatives) well. Because they know that we have felt their pain. Each one. Because if you meet such a family, initially it may be so chaotic and so complicated. And so many ugly words or yelling or whatever. That makes it hard to, sort of, put up with it and think well of them. And I think that the alliance sessions affect us somehow. In the way we approach them. I think that with all the families I have worked with in that way, I have a completely different relationship than with other patients and their relatives.’

Family involvement gave clinicians increased access to collateral information, which contributed significantly to their understanding of the patient, in terms of clinical history, warning signals and the resources and capabilities that the patient had possessed before getting ill. Clinicians also gained insight into the patient’s context, including social relations and interactions, which afforded them a more holistic view of the patient.

**FG13:** ‘(…) you do get, right (…) a different picture of the patient (…) that sorrow and joy of how life both was and how in a way life has changed (…) Because it, it has something to do with being able to perhaps see some other possibilities in the patient.’

#### 3.1.4. Being on the same team

The final theme identified in ‘the triad’ was that family involvement generated a sense of ‘being on the same team.’ It meant acknowledging relatives as valuable partners and that clinical assessment, treatment and follow-up was a collaborative effort, where patient, relative(s) and clinician(s) pulled in the same direction as allies. Clinicians described this feeling of being on the same team as an antidote to the loneliness that both patient and relative may experience, in dealing with the illness on their own.

**FG2**: ‘(…) and that they (the relatives) feel that they have a supportive role, that we are on the same team in a way. That everybody wants the best outcome, for instance not to have a new hospital admission (…), rather than it being «my responsibility, me alone, I am the one who is ill, I have to carry the burden», then it is more of a community around it.’

Clinicians recognised that it was vital to have an open line of communication, preferably by establishing contact with relatives early and in a calm phase, rather than late and during an acute crisis (which had been the norm). An open line meant that relatives had the possibility to contact clinicians directly for guidance and support, which appeared to reduce relatives’ stress significantly. It could also mean that, with the patient’s consent, clinicians would contact relatives for a mutual update, which increased the quality of follow-up.

**FG4:** ‘So what I like about it is that relatives have… Have an open line (of communication) with me. That I become a person who it is possible to reach without it… Without them having to jump through several hoops. To obtain special permits and such. One sort of gets that collaboration established and then it is there during a worse phase, then you sort of have a… A safety net (…).’

The quote above further illustrates how, by having an open line, relatives could perform an essential role as a safety net. With increased understanding of symptoms and warning signals, relatives were capable of detecting clinical deterioration earlier and alerting the health services, particularly when involved in critical treatment decisions and plans for crisis management.

### 3.2. Perceived disadvantages or challenges of family involvement

When asked directly about disadvantages or challenges of family involvement, many clinicians reported that they had experienced few or none. The three main themes in this section constitute a synthesis of the most frequently described disadvantages or challenges. However, clinicians did not always consider these challenges unequivocally negative when placed in their proper context. Two additional main themes were left out of this article due to potential overlap with previous publications. These were ‘Challenges related to contact, confidentiality and information sharing with relatives’ (38) and ‘FPE is resource demanding’ (34) (Figure 2).

In addition to the main themes, several codes were identified in only 1–2 focus groups, indicating a significant variety in the perception of and experiences with these challenges. Examples include that information could scare relatives or make them feel guilty, that clinicians were afraid of ‘infantilising’ the patient by involving relatives or that FPE, with its fixed schedule and communication rules, could be experienced as artificial or restrictive.

#### 3.2.1. Family psychoeducation—occasional poor model fit or difficulties following the framework

Some clinicians reflected that the FPE model was most appropriate for younger and recently diagnosed patients, and that the training mainly focused on patients living with their parents. Although the model could address common reactions, issues and dysfunctional patterns in a family with a mentally ill person, there were also instances of poor model fit when the patient was too ill or the family conflicts too severe. In such cases, clinicians frequently described FPE as insufficient, and how following the structure could be difficult or unsuitable.

**FG1:** ‘(…) That… They (the relatives) should have an increased understanding of (…) the patient. In FPE, the patient does have a bit… Yes, is in charge a little. In this particular case, I experience them as a deeply traumatised family after a lot of… Ehm… Problematic behaviour on the part of the patient. Where I feel that we fall short, with our current measures (FPE) (…).’

#### 3.2.2. Getting more involved than usual

Clinicians recognised the benefits of creating a space for relatives’ experiences, emotions and needs, as well as offering them adequate support. However, they also described how there was a thin line between this practice and becoming the relative’s therapist. They sometimes struggled to determine the limits of their responsibility for relatives’ health and well-being, particularly if the relatives were suffering from mental illness themselves.

**FG3:** ‘(…) Because it has happened, that the patient was completely out of focus and it was all about mother’s needs (…) Then you have to set limits and… Strict limits as well.’

Clinicians would also feel the despair, sorrow and pain of relatives more directly, with the risk of getting too emotionally involved and loosing professional distance. However, it was recognised as an unavoidable part of involving relatives and letting them share their experiences and emotions, and clinicians considered that the benefits outweighed this particular disadvantage.

**FG6:** ‘That is because the patient is so ill. And then there is also the fact that we, in such situations, may become co-sufferers. That we feel the emotional part, the despair and hopelessness that the family experiences and become slightly infected by it (…).’

The chance to observe social interactions within the family and to understand the patient’s context was considered invaluable. However, with this position and knowledge clinicians also felt that the scope of their responsibility widened, and that they suddenly played a role in family dynamics.

**FG8:** ‘I think it is a dilemma (…) that we support the family, but perhaps what is needed is a separation. That is to say, the patient who is 34 years old has to move out soon maybe, and the dilemma is to what extent should we hold an opinion about that?’

#### 3.2.3. Relatives as a potentially negative influence—important nonetheless

Clinicians described how relatives might constitute a negative influence on the patient in two main ways. Firstly, some relatives disagreed with clinicians about diagnosis and/or treatment, despite efforts to establish a shared understanding. Many went to file complaints against the services and clinicians were afraid that the relatives would sabotage the patient’s treatment. They observed that adherence to treatment was often compromised when relatives were not onboard.

**FG2:** ‘(…) And where the patient suffers and, or they are caught in between often, (…) I think many of them experience too (that) maybe we and (their) relatives disagree right. That relatives evaluate our treatment, medication, that it is not good, (it) does not help the trust and (therapeutic) relationship we are working on at the policlinic (…) Patients with psychosis do not tolerate it very well.’

However, clinicians did not consider that differing opinions was an argument against family involvement. On the contrary, it was important to explore their expectations and views through having contact.

Secondly, clinicians described how relatives might constitute a negative influence directly on the patient by being critical, overinvolved, neglectful or unable to understand despite participating in structured family involvement. An important realisation among several clinicians was that the family may not be ideal or even a particularly good influence, but it is still important to the patient. Consequently, family involvement is nearly always required to understand how the family works and help them adjust if possible or, as a last resort, help the patient to maintain some distance to the relatives.

**FG5:** ‘(…) And I still have that thought in the back of my mind, that one grew up with this family and often perhaps they did not do the right things, but I have nonetheless adjusted my thoughts concerning the family. That is, the family is a part of, it may be a part of the problem, but at the same time it may be a part of the solution.’

## 4. Discussion

To the clinicians in this study, involving the family meant that patients were not alone in dealing with their illness, relatives were not alone with their burden and concerns, and clinicians were not alone in doing clinical assessments and follow-up.

Through their accounts, we see how the central benefits of family involvement in the treatment of psychotic disorders can be viewed as an interconnected triad. Reducing conflict and stress, a triadic understanding and being on the same team appeared to be mutually reinforcing themes in a continuous process. Furthermore, this triad of benefits was linked to three important clinician-facilitated sub-themes: a space for relatives’ experiences, emotions and needs; a space for patients and relatives to discuss sensitive topics and; an open line of communication between clinician and relative to ensure appropriate follow-up and continuous support.

### 4.1. Perceived benefits of family involvement

As mentioned previously, qualitative studies on clinicians’ perceptions of FPE and similar interventions have mainly focused on barriers and challenges. However, consistent with our findings, they also report that health professionals generally consider the framework and tools useful, while emphasising the need for flexible adaptations (24, 25, 41).

Some qualitative studies have investigated patients and relatives’ perspectives on FPE and similar interventions in single- and/or multi-family formats. These often emphasise how improved communication and a reframing of relatives understanding leads to a reduction in conflict and stress (18, 19, 21). Their findings resonate well with the perceived benefits and processes identified in our study, and are consistent with the theory that FPE generates a reframing of relatives understanding, which through a reduction in the level of EE leads to reduced relapse rates (17). The clinicians in our study emphasised how negative symptoms were particularly hard for the relatives to identify as being part of the illness, and thus vital to address in order to achieve this reframing. They also reported that increased understanding among relatives might lead to better monitoring and follow-up of the patient.

Qualitative studies of family interventions have also described increased mutual understanding within the family, as well as increased family cohesion and unity among some participants (22, 23). However, a contribution of this study is to describe how mutual understanding and acknowledgement between all three stakeholders may increase during family involvement. Our findings also suggest that a shared understanding, of illness-related concepts and processes, is linked to increased mutual understanding and acknowledgement through a mutually reinforcing process. We therefore use the term ‘a triadic understanding’ to describe both shared and mutual understandings and their reciprocal connections.

A triadic understanding may be accompanied by a sense of ‘being on the same team.’ Previous qualitative studies of multi-family interventions have emphasised the importance of peer support and a sense of belonging (19, 20, 22). Still, our data indicate that a reduced feeling of loneliness, as well as an increased sense of belonging and inclusion, may be important mediators of single-family interventions as well. Qualitative studies of general family involvement in mental health care have described how clinicians may consider relatives valuable partners, teaming up with them to provide the patient with the best possible care (28, 42–45). Yet our findings demonstrate how clinicians may expand the concept of ‘being on the same team’ to include the patient as well.

The FPE and BFIS models may provide clinicians, relatives and patients with a basis for achieving this triad of benefits together, but through clinicians’ accounts we may also recognise their critical role in this process. Creating a space for relatives’ experiences, emotions and needs; a space for patients and relatives to discuss sensitive topics and; an open line of communication between clinician and relative, may be regarded as important clinician-facilitated elements to establish and maintain successful family involvement. These sub-themes were directly linked to a reduction in conflict and stress during all phases of family involvement, by our participants. Qualitative studies of general family involvement in mental health care also emphasise that relatives should be offered a space by themselves to share their experiences, emotions and needs (26, 28, 45–47), as well as the importance of having an open line of communication to ensure continuous support, appropriate follow-up and enabling relatives to act as a safety net (28–30, 43–45). However, perhaps the most prominent theme in qualitative research on family involvement is how it leads to increased understanding and acknowledgement, when there is a space to share experiences and discuss sensitive topics, characterised by openness, trust and support. This appears to be the case, regardless of whether researchers have explored family involvement in general or specific interventions, whether it was offered in single- or multi-family format and whether it was grounded in a biopsychosocial or postmodern ethos (18, 19, 22, 23, 27, 44, 46, 48, 49). A recent review (17) similarly found that common therapeutic factors—therapeutic alliance, support and the opportunity for sharing—might contribute significantly to the effects of family interventions, in a manner already recognised in psychotherapy research.

### 4.2. Perceived disadvantages or challenges of family involvement

To succeed with the implementation of family involvement in mental health care, it is vital to acknowledge the disadvantages or challenges that clinicians may experience. In the IFIP trial, lack of shared perceptions, competence, routines, resources and uncertainty regarding the engagement phase and confidentiality were identified as major barriers (34, 38). The present study however, looked at perceived disadvantages or challenges in clinical practice when family involvement actually takes place.

Clinicians reported disadvantages or challenges less frequently, and with a larger variety, than they described the benefits of family involvement. This might indicate that random factors related to particular patients, families, sites or clinicians, rather than family involvement itself, could explain some of the challenges.

The fact that the FPE model and its structure does not fit every client and relative is generally recognised (25). However, it is interesting that clinicians with extensive experience with FPE seem to consider that there is poor model fit for some patients or families (25, 41), while clinicians in a study who received training but did not practise FPE considered the model to be unfit for most of their patients (24). This may corroborate the findings of a previous article from the IFIP trial, which showed that practicing family involvement and experiencing its utility first-hand, for all stakeholders in various situations, is important for clinicians to overcome central barriers (34).

The challenges with getting more involved than usual were described as inevitable by clinicians, who in general considered the benefits to outweigh the disadvantages. However, a prominent finding was that they were conscious not to become the relative’s therapist, a notion that has been reported in previous studies (42, 50). This dilemma perhaps exemplifies a more general problem in the health and care services, which is the uncertainty as to who should look after relatives’ health and well-being. It may also reflect how the education of health professionals in Norway has traditionally focused on the patient, without adequately emphasising the importance of relatives and the patient’s social network.

Finally, we see how clinicians realised that family involvement was nearly always required and useful. If relatives disagreed with the diagnosis or treatment, or if they constituted a potentially negative influence on the patient in other ways, it was necessary to uncover this through family involvement and act accordingly. This constituted a major shift in clinicians’ attitudes, from primarily focusing on disadvantages and barriers to recognising these obstacles but emphasising solutions and benefits instead (34).

### 4.3. Strengths and limitations

Being nested in a successful implementation trial (37), the present study is in a unique position to explore how clinicians perceive the benefits of family involvement when major barriers to implementation on both organisational and clinical levels have been traversed (34, 51, 52). Similarly, it can give an outline of the disadvantages or challenges that clinicians nonetheless experience.

Many of the perceived benefits and processes identified in the present article have been described in previous qualitative studies on patients and relatives’ perspectives, which may corroborate our findings. This study is also likely the first to explore all of these perceived benefits together and to place them in a thematic ‘system’ according to the processes described by clinicians. It is probably made possible by two main factors. Firstly, implementing BFIS together with FPE makes this study uniquely placed to explore the dynamics between basic and complex forms of family involvement. However, it also means that it cannot be regarded as a ‘pure’ exploration of the mediating factors of the FPE model.

Secondly, by focusing on clinicians’ perspectives we get a ‘bird’s-eye view’ of benefits for both patients, relatives and clinicians, with certain limitations. Unlike previous studies on patients’ perspectives for instance, the clinicians in our study did not emphasise increased coping skills (22), personal responsibility or independence (18, 23) for the patient. Another example concerns the theme ‘a triadic understanding,’ where clinicians were less concerned with patients understanding relatives better. However, we know from the analysis of interviews with patients that understanding relatives’ perspective and situation was an important part of their experience (Hansson et al., submitted). It shows how perspectives from patients and relatives are needed to complement the findings, which is why the IFIP trial conducted qualitative studies on relatives and patients’ experiences as well.

Clinicians did not report their experiences unfiltered and their narratives might be shaped by their training and knowledge of how the FPE model is supposed to work. Similarly, the normative position and theoretical knowledge of the researchers might have influenced the interpretation of clinicians’ accounts, possibly adding a second layer of confirmation bias. It is possible that focus groups with clinicians in the control arm or in units that refused to participate would have provided us with other perspectives. Although we strived to include critical voices in this study, it is possible that ‘dissidents’ did not participate, either through their own choice or the local manager’s decision. Even if they did participate, it is possible that they did not feel able to speak their mind freely in front of colleagues and researchers. This also points to a general limitation of the focus group format.

The topics discussed in this study concern the relationships and dynamics between elements of family involvement and observed benefits or disadvantages, but the qualitative methodology is not suitable to investigate causality. We must therefore regard this as an explorative study that may generate hypotheses for quantitative research. In terms of external validity, our study took place in a specific geographical, clinical and cultural context, and focused only on family involvement for persons with psychotic disorders. However, there is increasing evidence that these interventions are relevant to other patient groups (53, 54), as well as in other sociocultural contexts (55).

### 4.4. Implications

Our findings might indicate that implementing BFIS and FPE together may be particularly advantageous. They further seem to warrant a particular emphasis on negative symptoms during psychoeducation and an increased awareness of the important clinician-facilitated elements during all phases of family involvement. Perceived disadvantages or challenges should be acknowledged and addressed in future implementation efforts and research.

## 5. Conclusion

This nested qualitative study showed how clinicians mainly reported positive experiences with family involvement in the treatment of psychotic disorders. The FPE model and framework was experienced as particularly useful. Family involvement led to a ‘triad’ of perceived benefits: reducing conflict and stress, a triadic understanding and being on the same team. Clinicians further facilitated this triad of benefits by creating a space for relatives’ experiences, emotions and needs, a space for relatives and patients to discuss sensitive topics and an open line of communication with relatives to provide continuous guidance and support. The challenges described were occasional poor model fit, being involved more than usual and that relatives might constitute a negative influence on the patient. Our findings could be used to inform clinical practice, as well as future quantitative research on mediating factors and implementation efforts.

## Data availability statement

The datasets used and/or analysed during the current study are not available, since the participants of this study did not give written consent for their data to be shared publicly.

## Ethics statement

The studies involving human participants were reviewed and approved by the Norwegian Regional Committee for medical and health research ethics (REC) South-East, registration number 2018/128. The patients/participants provided their written informed consent to participate in this study.

## Author contributions

All authors made significant contributions to the conception and design of the study, with particularly substantial contributions from KSH and RP. MR did a preliminary mapping of the participating units’ structure, organisation and practice, and made substantial efforts to recruit clinical units with aid from the other authors. Data collection was performed by all the authors. Analysis was mainly carried out by LH, with assistance from RP, MR, and KMH. LH wrote the first draft of this article. All the authors critically revised the article, gave their final approval before submission, and agreed to be accountable for all aspects of the work in ensuring that questions related to the accuracy or integrity of any part of the work are appropriately investigated and resolved.

## Funding

The study was funded by the Research Council of Norway, grant number: 262863. This funding source had no role in the study design, execution, analyses, interpretation of the data, the writing of manuscripts or decision to submit results.

## Conflict of interest

The authors declare that the research was conducted in the absence of any commercial or financial relationships that could be construed as a potential conflict of interest.

## Publisher’s note

All claims expressed in this article are solely those of the authors and do not necessarily represent those of their affiliated organizations, or those of the publisher, the editors and the reviewers. Any product that may be evaluated in this article, or claim that may be made by its manufacturer, is not guaranteed or endorsed by the publisher.

## Supplementary material

Supplementary DATA SHEET 1 SRQR checklist.

Supplementary DATA SHEET 2 Interview guides.
