# Supplementary material for: Clinicians’ perceptions of family involvement in the treatment of persons with psychotic disorders: a nested qualitative study
Source: Front Psychiatry. 2023 May 24;14:1175557. doi: 10.3389/fpsyt.2023.1175557 (PMC10244542; doi:10.3389/fpsyt.2023.1175557)
Supplement: Supplementary DATA SHEET 2 — Interview guides. [file Data_Sheet_2.PDF]

Supplementary Material 2 – Interview guides

Interview guide – Focus groups with implementation teams

Overview of topics to be covered during the focus groups

Remember to ask for specific examples

Intro: Summarise the most important changes at the unit on all levels, check whether this information is correct, and then focus initially on the significance of the services that are increasingly offered to patients and relatives.

The significance of improved family involvement practices when in contact with patients and relatives (The clinical elements of the IFIP intervention: conversations, written information material, psychoeducative seminars for relatives, and family psychoeducation)

- For the patients.
- For the relatives.
- For yourself as health professionals, and the services.
- Is there anything else they should be offered?

Ethical dilemmas and conflicts of interest during family involvement, and other barriers and facilitators

- Which dilemmas/conflicts of interest have you experienced during family involvement, at the unit? (Patient vs. relatives. What roles do the clinicians' perceptions and interests play?)
- What challenges have you experienced concerning the exercise of the duty of confidentiality during family involvement?
- What challenges have you experienced with regard to receiving/documenting information from relatives?
- How were these situations handled? Could anything have been done otherwise?
- Hand out the 'barrier and facilitator' document and ask them to comment on any missing factors.
- Which measures could be useful at the administrative and policy level (health trust/national level)? (For instance legislation, financial incentives, documentation systems, more clearly stated policies on next of kin).

Experiences with the implementation effort (local implementation team, family coordinator, training, and guidance)

- The effort to implement the IFIP intervention. What works well or not so well at your unit? Possible changes? Any suggestions for other measures?
- Experiences with the implementation support program? Positive and negative experiences? Any suggestions for changes?
- Is everybody in the unit committed to the project? Understanding of responsibility: What role and responsibility towards next of kin do you consider yourself to have, as health professionals? (Are there variations related to professional background?). Any changes?
- What impact do the clinical pathways for mental health and substance abuse have on the way you practice family involvement today?
- If we have time: Standardisation versus professional autonomy – what is a good balance?

Interview guide – Focus groups with ordinary clinicians

Overview of topics to be covered during the focus groups

Remember to ask for specific examples

Introduction: What are the most important changes that have taken place at your unit since the project began?

- How do you notice these changes in your daily work?
- Increased competence/assurance?
- Altered ways of thinking/attitudes?
- Altered ways of working?
- Changes in the services offered to the unit's patients and their relatives?

The significance of improved family involvement practices when in contact with patients and relatives (The clinical elements of the IFIP intervention: Conversations, family psychoeducation, crisis/coping plan, written information material, psychoeducative seminars for relatives). Positive and negative experiences. Ask a general open-ended question first, and then it is possible to ask specifically about each single element.

- For yourself as health professionals.
- For the health services.
- For patients and relatives.
- Specific examples.
- Any feedback from patients and relatives?
- Possibly mention the most important documented effects of family interventions and inquire whether they have experienced these effects.
- What has worked well at their unit, and why.
- What has not worked well at their unit, and why. Ask specifically about any suggestions for changes.
- Is there anything else that patients and relatives should be offered?

Challenges related to the duty of confidentiality and documentation

- Quite a few health professionals report that they face challenges related to the duty of confidentiality during family involvement. Have you experienced such challenges? Any changes?
- During the IFIP project, we have experienced that many clinicians are unsure of where and how they should receive/document information from relatives. Have you experienced such uncertainty? Any changes?
- (*If they report challenges*) How were these situations handled? Could anything have been done otherwise?

The significance of competence development and improved structure of family involvement practices (procedures and routines, documentation, family coordinator, systematic assessments of FPE eligibility etc.) and tools/resources (e.g. the conversation guide). Positive and negative experiences. Ask a general open-ended question first, and then it is possible to ask specifically about each single measure.

- For yourself as health professionals.
- For the health services.
- Which measures/tools have worked well at their unit? Why?
- Which measures/tools have not worked well at their unit? Why? Ask specifically about any suggestions for changes.
- Are there any measures/tools that we have not prioritised, which could have been useful?

Shared understanding, leadership commitment, and the clinical pathways

- Does the local context affect these factors?
- Would you say that there is a shared understanding of why and how one practices family involvement at the unit? If so, how does this shared understanding manifest itself?
- What role would you say the leadership at the unit has played in the project/implementation work? How does this affect the implementation work?
- What impact do the clinical pathways for mental health and substance abuse have on the way you practice family involvement today?

Experiences with the coronavirus pandemic

- How would you say that the coronavirus pandemic has affected your daily work?
- Has the follow-up of patients and relatives changed? If that case how?
- Have there been challenges with the family involvement during the pandemic? Examples? If yes, how were these challenges dealt with?
- Are there any of the measures implemented as part of the IFIP trial that have worked particularly well during this crisis? Are there any of the measures that have worked poorly? Examples?
- Are there any other family involvement measures that could have been useful in relation to the crisis? Examples?
